# Relationship Between Job Burnout and Neuroendocrine Indicators in Soldiers in the Xinjiang Arid Desert: A Cross-Sectional Study

**DOI:** 10.3390/ijerph121214977

**Published:** 2015-12-01

**Authors:** Ning Tao, Jianjiang Zhang, Zhixin Song, Jinhua Tang, Jiwen Liu

**Affiliations:** 1 Department of Public Health, Xinjiang Medical University, Urumqi 830011, China; zflningning@sina.com (T.N.); inforsong@163.com (Z.S.); tjh4362964@126.com (J.T.); 2 Center for Disease Prevention and Control of Xinjiang Command, Urumqi 830001, China; mnt2007@163.com

**Keywords:** job burnout, harsh environment, soldiers, neuroendocrine indicator

## Abstract

The purpose of this study was to explore the relationship between job burnout and neuroendocrine indicators in soldiers living in a harsh environment. Three hundred soldiers stationed in the arid desert and 600 in an urban area were recruited. They filled in the Chinese Maslach Burnout Inventory questionnaire. One hundred soldiers were randomly selected from each group to measure their levels of noradrenaline, serotonin, heat shock protein (HSP)-70, adrenocorticotropic hormone, and serum cortisol. Job burnout was more common in soldiers from urban areas than those from rural areas. Job burnout was significantly higher among soldiers stationed in the arid desert than those in urban areas. For soldiers in the arid desert, the levels of HSP-70, serum cortisol, and adrenocorticotropic hormone were significantly higher than in soldiers in urban areas. Correlation analyses showed that the degree of job burnout was weakly negatively correlated with the level of HSP-70. Being an only child, HSP-70 levels, cortisol levels, and ACTH levels were independently associated with job burnout in soldiers stationed in the arid desert. A higher level of job burnout in soldiers stationed in arid desert and a corresponding change in neuroendocrine indicators indicated a correlation between occupational stress and neurotransmitters.

## 1. Introduction

Job burnout is a state of stress that is an important issue worldwide since it has a direct impact on the efficiency and quality of work [1,2]. Job burnout can also influence physical and mental health with symptoms of mental exhaustion, physical fatigue, detachment from work, and feelings of diminished competence [3]. Soldiers are one of the high-risk groups for job burnout because they have more occupational responsibility and stress [4,5,6]. Studies in China have revealed that the prevalence of job burnout in the military was 88.14%, which was higher than the prevalence seen in teachers (14.49%) and nurses (69.1%) [7,8]. However, most of the symptoms of military job burnout were mild or moderate [7,8].

Consensus has been reached that job burnout mainly results from changes in the neuroendocrine system, in which excessive activation of the hypothalamus-pituitary-adrenal (HPA) axis leads to neuroendocrine and homeostasis disorders, with alteration of various hormones, neurotransmitters, and cytokines associated with the HPA axis [9,10]. Previous studies have shown that neuroendocrine indicators are important transmitters of molecular information between the immune and neuroendocrine systems, and that they are involved in the process of job burnout [11,12,13,14,15]. Monitoring of the neuroendocrine system is also important because if the stress response is long term this can result in acute and chronic illness [16].

In arid deserts and other harsh environments, people’s physical and mental health is affected by the natural conditions and monotonous lifestyle [17,18,19]. Kong *et al.* [20] found that there were significant differences in physiological and biochemical indexes between soldiers stationed in an arid desert and those living in a normal environment, indicating that the former has an impact on the physical health of soldiers. A soldier in a harsh environment can easily become mentally stressed, and long-term stress leads to an increase in norepinephrine, dopamine, and serotonin levels [21].

Therefore, the aim of the present study was to obtain preliminarily data about the prevalence of job burnout in soldiers stationed in the arid desert of Xinjiang, and to investigate the relationship between military job burnout and neuroendocrine indicators. This will provide important information on the risk factors for job burnout in soldiers in a harsh environment and may help develop methods to prevent this form of stress.

## 2. Experimental Section

### 2.1. Participants

This was a prospective study performed from April to July 2013. One battalion stationed in the arid desert region of Xinjiang was selected as the observation group and two battalions stationed in the urban area of Xinjiang were selected as the control group. All included soldiers had to have been enlisted for at least one year. Subjects using tricyclic antidepressants, hexadecadrol, estrogens, or glucocorticoids within 1 week to enrollment were excluded. The arid group comprised field operation personnel that were stationed in the desert as their main job. This was not a tour of duty but rather a long term placement. They worked five days on and three days off. This group could expect an increase in wages and full support in terms of articles required for daily use to compensate for their heavier work load. This study was approved by the Ethics Committee of the First Teaching Hospital of Xinjiang Medical University. Written informed consent was obtained from all participants.

### 2.2. Chinese Maslach Burnout Inventory (CMBI)

The CMBI questionnaire includes three factors: emotional exhaustion, depersonalization, and reduced accomplishment. Emotional exhaustion mainly refers to the weary and worn-out states that results from physical and emotional depletion; it is complicated in particular with emotional symptoms like fatigue, exhaustion, and worrying that one’s work will affect their own emotional states. Depersonalization mainly reflects individual’s poor social relationship and their negative, indifferent, and evasive attitudes towards their work, which include manifestations like indifference to the feelings of their subjects, blaming their subjects, and refusing their subjects’ requirements. Reduced accomplishment mainly indicates individual’s negative self-assessment of their work achievements, such as a sense of incompetence, and a lack of efficiency, morale, and achievement in their work.

Each factor consists of five items, for a total of 15 items. The questionnaire used seven magnitudes to score each item: 1 representing “completely fitting” and 7 representing “completely unfitting”. The dimension of reduced accomplishment (items 3, 6, 9, 12 and 15) used reverse scoring.

Four levels of job burnout were determined based on the cut-off values (emotional exhaustion score ≥25, depersonalization score ≥11, and reduced accomplishment score ≥16): no burnout (scored lower than the cut-off values in all three scales), mild burnout (scored no lower than the cut-off value in any one scale), moderate burnout (scored no lower than the cut-off values in any two scales), and severe burnout (scored no lower than the cut-off values in all three scales). This questionnaire and the cut-off values have been shown to be valid and reliable [22].

### 2.3. Quality Control of the Questionnaires 

All questionnaires were handed out and retrieved in the activity center. The questionnaires were completed anonymously within 20 min. Prior to the survey, soldiers were mobilized by two experienced psychologists and two officers to ensure the cooperation with the research team and to ensure the authenticity and effectiveness of the questionnaire filling. Study staff explained how to fill out the questionnaires, explained the methods and significance of the study, and ensured that the soldiers cooperated positively with a scientific and down-to-earth attitude so that they could honestly complete every item of the questionnaire. During the survey and after recording the results, all contents were comprehensively checked by the researchers, making sure any doubts about the responses could be inquired into and reconfirmed, mistakes corrected, and omissions completed.

### 2.4. Physiological and Biochemical Indexes 

One hundred soldiers who correctly filled out their questionnaire were selected from each group using simple random sampling to determine the neuroendocrine indicators (noradrenaline, serotonin, heat shock protein (HSP)-70, adrenocorticotropic hormone (ACTH), and serum cortisol). Fasting blood samples (5 mL) were obtained with the study subjects in the decubitus position. The blood samples were placed in heparin-anticoagulant tubes and centrifuged at 3000 rpm for 5 min at 4 °C. Plasma was collected and stored at −20 °C. Participants were asked to refrain from a high-fat diet and alcohol for three days before blood sampling. Levels of heat shock protein (HSP-70), noradrenaline, and serotonin were measured by ELISA (ADL, USA), and the levels of cortisol and ACTH were measured by radioimmunoassay (Beijing North TZ-Biotech Develop, Co., Ltd., Beijing, China), using a GC-2016 16-probe γ-radioimmune counter.

### 2.5. Statistical Analysis

The survey results were independently entered into the database by two researchers and a consistency test was conducted. SPSS 17.0 (IBM, Armonk, NY, USA) was used for data processing and statistical analysis. Results are presented as mean ± standard deviation or as frequencies, as appropriate. Continuous data were compared using the Student’s t-test. Categorical data were analyzed using the chi-square test. The Pearson correlation test was used to analyze the correlations between job burnout and neuroendocrine indicators. Multivariate logistic regression was used to identify the factors independently associated with job burnout including age, length of military service, ethnic background, education level, marital status, location of household registration before recruitment, monthly family income, rank, and neuroendocrine response. *p*-values < 0.05 were considered to be statistically significant.

## 3. Results and Discussion

### 3.1. Results

#### 3.1.1. Characteristics of the Participants

Three hundred soldiers were enrolled in the arid desert group and 600 soldiers were enrolled in the control group. Nine hundred questionnaires were distributed, and 820 (246 from the desert group (82.0%), and 574 (95.7%) from the urban group, *p* = 0.028) were retrieved and validated as being satisfactory, for a total retrieval rate of 91.1%. The age of the soldiers ranged from 16 to 44 years, with an average of 21.4 ± 3.3 years, without difference between the two groups (*p* = 0.096). The duration of military service ranged from 1 to 26 years, with an average of 3.1 ± 3.0 years, without difference between the two groups (*p* = 0.094) (Table 1).

The degree of military job burnout differed significantly with primary area of residence (*p* < 0.001). The degree of military job burnout (*p* < 0.001), reduced achievement (*p* = 0.001), and emotional exhaustion (*p* < 0.001) were significantly higher in the arid desert group than in the control group (Table 2). This suggests that the arid desert had an impact on job burnout, emotional exhaustion, and reduced achievement in soldiers.

**Table 1:** Characteristics of the participants.

	All Participants			Biochemistry Subset		
Variables	Arid Desert Group n = 246	Control Group n = 574	*p*	Arid Desert Group n = 75	Control Group n = 96	*p*
Age			0.096			0.473
≤20 years	122, 49.6%	241, 42.0%		38, 50.70%	40, 41.70%	
21–25 years	104, 42.3%	268, 46.7%		30, 40.0%	47, 48.90%	
≥26 years	20, 8.1%	65, 11.3%		7, 9.30%	9, 9.40%	
Length of military service			0.094			0.481
≤2 years	168, 68.3%	363, 63.2%		51, 68.0%	61, 63.5%	
3–7 years	62, 25.2%	146, 25.4%		20, 26.7%	25, 26.0%	
≥8 years	16, 6.5%	65, 11.3%		4, 5.3%	10, 10.4%	
Ethnicity			<0.001			0.022
Han	235, 95.5%	495, 86.2%		72, 96.0%	82, 85.4%	
Minority	11, 4.5%	79, 13.8%		3, 4.0%	14, 14.6%	
Education			0.971			0.995
Junior and senior	179, 72.8%	414, 72.1%		53, 70.7%	68, 70.8%	
Big and secondary	53, 21.5%	128, 22.3%		16, 21.3%	20, 20.8%	
University or above	14, 5.7%	32, 5.6%		6, 8.0%	8, 8.3%	
Marital status			0.008			0.076
Married	11, 4.5%	58, 10.1%		2, 2.7%	9, 9.40%	
Unmarried	235, 95.5%	516, 89.9%		73, 97.3%	87, 90.60%	
Household registration before recruitment			<0.001			0.425
Urban	194, 78.9%	370, 64.5%		55, 73.3%	65, 67.7%	
Rural	52, 21.1%	204, 35.5%		20, 26.7%	31, 32.3%	
Only child			0.581			0.787
Yes	69, 28.0%	172, 30.0%		22, 29.3%	30, 31.3%	
No	177, 72.0%	402, 70.0%		53, 70.7%	66, 68.8%	
Monthly family income			0.636			0.801
≤1000 yuan	27, 11.0%	78, 13.6%		8, 10.7%	12, 12.5%	
1000–4000 yuan	34, 13.8%	91, 15.9%		11, 14.7%	16, 16.7%	
4000–7000 yuan	46, 18.7%	107, 18.6%		15, 20.0%	19, 19.8%	
7000–1000 yuan	91, 37.0%	185, 32.2%		29, 38.7%	29, 30.2%	
≥10,000 yuan	48, 19.5%	113, 19.7%		12, 16.0%	20, 20.8%	
Rank level			0.438			0.918
Officer	11, 4.5%	31, 5.4%		3, 4.0%	5, 5.2%	
Sergeancy	68, 27.6%	180, 31.4%		22, 29.3%	29, 30.2%	
Conscripts	167, 67.9%	363, 63.2%		50, 66.7%	62, 64.6%	

* *p* < 0.05.

**Table 2:** Results of burnout and neuroendocrine indicators.

	All Participants			Biochemistry Subset		
Variables	Arid Desert Group n = 246	Control Group n = 574	*p*	Arid Desert Group n = 75	Control Group n = 96	*p*
Job burnout score	43.94 ± 14.61	41.20 ± 15.10	<0.001 *	42.58 ± 12.36	40.31 ± 14.08	<0.001 *
Emotional exhaustion	15.34 ± 7.95	13.59 ± 7.12	<0.001 *	16.11 ± 7.23	13.98 ± 7.35	<0.001 *
Depersonalisation	10.17 ± 5.61	9.60 ± 5.21	0.131	9.89 ± 5.12	9.54 ± 4.97	0.342
Reduced accomplishment	19.62 ± 7.92	19.00 ± 8.24	0.001 *	20.03 ± 7.98	19.12 ± 8.31	<0.001 *
Job burnout level			0.010 *			0.047 *
no burnout	49, 19.9%	144, 25.1%		16, 21.3%	25, 26.0%	
mild burnout	99, 40.2%	256, 44.6%		28, 37.3%	47, 49.0%	
moderate burnout	75, 30.5%	132, 23.0%		24, 32.0%	21, 21.9%	
severe burnout	23, 9.3%	42, 7.3%		7, 9.3%	3, 3.1%	
Neuroendocrine response						
HSP-70 (ng/L)				3.83 ± 1.93	3.67 ± 2.42	0.043 *
Noradrenaline (ng/L)				3.67 ± 2.88	3.49 ± 2.43	0.165
Serotonin (ng/L)				3.38 ± 2.74	2.05 ± 0.70	0.314
Cortisol (ng/mL)				167.94 ± 87.56	153.09 ± 49.70	0.012 *
ACTH (pg/mL)				22.61 ± 9.18	16.03 ± 6.40	<0.001 *

#### 3.1.2. Neuroendocrine Indicators

The levels of HSP-70 (*p* = 0.043), serum cortisol (*p* = 0.012), and ACTH (*p* < 0.001) were significantly higher in soldiers stationed in the arid desert than those in the urban areas (Table 2).

#### 3.1.3. Multivariate Analysis of Job Burnout in Soldiers in the Arid Desert

Job burnout was the dependent variable and individual subject characteristics (e.g., age and ethnicity) and related neuroendocrine indicators were entered as independent variables. Multiple regression analysis showed that being an only child (OR = 0.394, 95% CI: 0.174–0.891, *p* = 0.025), HSP-70 levels (OR = 1.740, 95% CI: 1.459–2.053, *p* = 0.022), cortisol levels (OR = 1.124, 95% CI: 1.045–1.206, *p* = 0.041), and ACTH levels (OR = 1.316, 95% CI: 1.127–1.532, *p* = 0.033) were independently associated with job burnout (Table 3).

**Table 3:** Multivariate analysis of job burnout in soldiers stationed in the arid desert.

	OR	95% CI	*p*
Only child	1.171	(1.032−1.874)	0.039 *
HSP-70	1.740	(1.459−2.053)	0.022 *
Cortisol	1.124	(1.045−1.206)	0.041 *
ACTH	1.316	(1.127−0.532)	0.033 *

* *p* < 0.05.

#### 3.1.4. Correlation between Job Burnout and Neuroendocrine Indicators 

There were weak correlations between HSP-70 levels and job burnout (r = −0.078, *p* = 0.011), cortisol levels and reduced accomplishment (r = −0.123, *p* = 0.002), and ACTH levels and reduced accomplishment (r = −0.126, *p* = 0.001) (Table 4).

**Table 4:** Correlation between job burnout and neuroendocrine indicators in the arid desert group.

Variables	Emotional Exhaustion	Depersonalization	Reduced Accomplishment	Job Burnout
HSP-70 (ng/L)	−0.045	−0.061	−0.031	−0.078 *
Cortisol (ng/mL)	0.011	0.06	−0.123 *	0.028
ACTH (pg/mL)	0.032	−0.072	−0.126 *	0.013

* *p* < 0.05.

### 3.2. Discussion

In the arid desert, soldiers find themselves in a tough natural environment, far removed from other people, traffic and communications, and they become a secluded population with uncommon characteristics. Furthermore, this particular special living environment deprives them of interpersonal interactions, and has monotonous cultural activities [23]. In addition, because they are stationed on the national borders, they are continuously faced with intense work and military training. There is a strong sense of organizational discipline in the military, which makes it easy for soldiers to become trapped in conditions of emotional stress, resulting in psychological dysfunction that can affect health and cause diseases [24].

Compared with soldiers stationed in urban areas, the degree of job burnout of soldiers stationed in the arid desert group was higher. The results suggest that special environments affect the degree of job burnout. These results are consistent with other studies showing that people living in harsh environments have a higher risk of developing mental illnesses [12,19].

The results of this study showed that the level of HSP-70, serum cortisol, and ACTH in the arid desert group were higher than in the control group, which is possibly due to the influence of dry climate, strong solar radiation, and evaporation on HSP-70 [24,25]. Indeed, peripheral chemoreceptors stimulated by harsh environments upregulate the release of adrenocorticotropic hormone by the hypothalamus and therefore an increase in ACTH secretion by the pituitary [9,10]. This can promote synthesis and secretion of adrenal cortex hormones, especially glucocorticoid-cortisol, resulting in significantly higher levels of serum cortisol and ACTH compared with the control group.

These changes in neuroendocrine factors are probably a response to the harsh working environment that results in job burnout and they may also lead to changes in the incidence of job burnout. Indeed, previous studies have shown that long-term stress and job burnout are associated with changes in neurotransmitters, neuropeptides, and steroidal stress mediators [11,14,20]. In the present study, there was a weak negative correlation between reduced achievement and the level of serum cortisol and ACTH, while job burnout was weakly negatively correlated with HSP-70. Multivariate analysis showed that being an only child, cortisol levels, ACTH levels, and HSP-70 levels were independently associated with job burnout, which is consistent with these previous studies.

The “little emperor syndrome” is an aspect of China’s one-child policy in which the only child attracts excessive amounts of attention from his parents. One-child families are more prevalent in urban areas. The child often receives too much love and has been highly mentally and physically restricted to devote himself to a heavy load of schoolwork, considering that the economic future of the family depends on his success. There is also evidence that many young Chinese feel heavily burdened and a huge sense of responsibility toward their parents, understanding that their success can have crucial consequences for their family [26]. In the present study, being an only child seemed to be a risk factor for job burnout in soldiers stationed in the arid desert. We also found that those subjects whose household before recruitment was in an urban area showed higher job burnout score than those from rural areas (data not shown), but “household registration before recruitment” was not a risk factor for job burnout after multivariate analysis. However, further study is necessary to address this issue.

This study showed no relationship between the ethnic group of the study subjects and job burnout score (data not shown). There is a suggestion that ethnicity may be an important factor because South Asians resident in the UK showed lower self-reported work strain than Europeans [27]. However, there were differences in the type of work that these populations undertook, so this may explain the ethnic differences [28]. In the present study the majority of the study population was of Han ethnicity (89%) so the minority ethnic group was quite small, with only 90 people. Therefore, we may not have had a large enough sample size to identify differences between the groups.

The present study is not without limitations. Indeed, the cross-sectional design prevented us to explore other causes of burnout. The sample size was small and from a single military region in China. Only a few markers of stress were measured, and more markers could be assessed in the future to obtain a more comprehensive picture. Only weak correlations were observed, which might be due to the small sample size of the subset of patients in whom biochemical tests were performed. In future we plan to undertake a cohort study to investigate occupational stress-induced hypertensive disorders of people exposed to the arid desert environment of Xinjiang. This will provide more evidence for the importance of understanding stress and job burnout in this military population.

## 4. Conclusions 

In conclusion, these results suggest a higher level of job burnout in soldiers stationed in arid desert and a corresponding change in neuroendocrine indicators, indicating a correlation between occupational stress and neurotransmitters.
